# Oncogenic regulation of tumor metabolic reprogramming

**DOI:** 10.18632/oncotarget.10911

**Published:** 2016-07-28

**Authors:** Míriam Tarrado-Castellarnau, Pedro de Atauri, Marta Cascante

**Affiliations:** ^1^ Department of Biochemistry and Molecular Biomedicine, Universitat de Barcelona, Institute of Biomedicine of Universitat de Barcelona (IBUB) and CSIC-Associated Unit, Barcelona, Spain

**Keywords:** metabolic reprogramming, MYC, HIF, PI3K, mTOR

## Abstract

Development of malignancy is accompanied by a complete metabolic reprogramming closely related to the acquisition of most of cancer hallmarks. In fact, key oncogenic pathways converge to adapt the metabolism of carbohydrates, proteins, lipids and nucleic acids to the dynamic tumor microenvironment, conferring a selective advantage to cancer cells. Therefore, metabolic properties of tumor cells are significantly different from those of non-transformed cells. In addition, tumor metabolic reprogramming is linked to drug resistance in cancer treatment. Accordingly, metabolic adaptations are specific vulnerabilities that can be used in different therapeutic approaches for cancer therapy. In this review, we discuss the dysregulation of the main metabolic pathways that enable cell transformation and its association with oncogenic signaling pathways, focusing on the effects of c-MYC, hypoxia inducible factor 1 (HIF1), phosphoinositide-3-kinase (PI3K), and the mechanistic target of rapamycin (mTOR) on cancer cell metabolism. Elucidating these connections is of crucial importance to identify new targets and develop selective cancer treatments that improve response to therapy and overcome the emerging resistance to chemotherapeutics.

## INTRODUCTION

Multifactorial diseases are the final result of the interaction between genetic susceptibility and environmental factors in which a clear hereditary pattern is not found. This complexity causes difficulties in the risk evaluation, diagnosis and treatment of these diseases. Cancer, one of the most prevalent multifactorial diseases, is characterized by the lost of physiological control and the malignant transformation of cells that acquire functional and genetic abnormalities, leading to tumor development and progression. In some cases, cancer cells have the ability to invade other tissues resulting in metastasis, the major cause of death from cancer. According to the most recent data released by the World Health Organization (WHO) in 2012, more than 14 million of new cancer cases were diagnosed, and 8.2 million cancer deaths and 32.4 million people living with cancer (within 5 years of diagnosis) were registered worldwide [1]. The most common cancers by primary site location were lung, prostate and colorectal in men, and breast, colorectal and cervix uteri in women [1].

Tumor cells present common biological capabilities sequentially acquired during the development of cancer that are considered essential to drive malignancy and known as the hallmarks of cancer [2]. These hallmark capabilities include sustaining proliferative signaling, evading growth suppressors, avoiding immune destruction, enabling replicative immortality, activating invasion and metastasis, inducing angiogenesis, resisting cell death and reprogramming cellular metabolism. In addition, there are two consequential characteristics of tumorigenesis that enable the acquisition of the hallmarks of cancer. The most prominent is the development of genomic instability and mutability, which endow tumor cells with genetic alterations that can orchestrate tumor progression. The second one involves the tumor-promoting inflammation by innate immune cells, which in turn serve to support multiple hallmark capabilities [2].

Non-transformed cells tightly regulate the mitogenic signaling that command cell growth and division in order to maintain a balance between cell proliferation and death. Accordingly, the dysregulation of the signaling pathways that regulate the progression through cell cycle, cell survival and metabolism may lead to malignant transformation. It is worth noting that neoplastic transformation requires not only the alteration of proliferative stimuli but also the disruption of mechanisms that prevent unrestrained proliferation such as programmed cell death (apoptosis) or negative-feedback signaling [3]. Likewise, the cooperative activation of oncogenes (genes that promote cell growth, proliferation and survival) and/or inactivation of tumor suppressor genes (genes that restrain cell growth and proliferation, promote DNA repair or trigger apoptosis) are involved in tumor development [3, 4]. Oncogenes can be activated through several mechanisms including upregulated transcriptional expression, increased stability of mutant proteins, altered functionality of proteins and abnormal recruitment or subcellular localization of gene products through interaction with aberrantly expressed or mutant binding partners [3, 5]. The products of oncogenes comprise transcription factors (e.g. c-MYC, hereafter referred to as MYC), growth factor receptors (e.g. EGFR), signal transduction proteins (e.g. RAS and PI3K), serine-threonine protein kinases (e.g. Akt, mTOR, CDK4 and CDK6) and inhibitors of apoptosis (e.g. BCL2) [5]. On the other hand, tumor suppressor genes encode proteins that inhibit cell division and cell proliferation (e.g. RB, p53, p16^INK4a^, PTEN), stimulate cell death (e.g. caspase 8 and p53) and repair damaged DNA (e.g. MSH2, MSH6, ATM and ATR) [6].

Accumulation of genetic alterations is associated with tumor evolution, which includes single nucleotide mutations and also whole-chromosomal changes [7–9]. In addition, epigenetic mechanisms including histone modifications, DNA methylation and non-coding RNAs are involved in carcinogenesis [10, 11]. In fact, tumors often display aberrant methylation patterns such as hypermethylation on the promoters of tumor suppressor genes causing transcriptional repression, and hypomethylation of oncogenes supporting their activation (reviewed in [11, 12]). Epigenetic modifications have been reported to regulate the Warburg effect and coordinate the overall cellular metabolism, including the pentose phosphate pathway and other pathways for sugar, lipid and amino acid metabolism, by affecting several metabolic enzyme activities [13–16]. Remarkably, oxidative stress is involved with both genetic and epigenetic modifications, playing an important role in carcinogenesis [10, 17].

## METABOLIC REPROGRAMMING OF TUMOR CELLS

Metabolism is the term that is used to describe the integrated network of chemical reactions involved in sustaining growth, proliferation and survival of cells and organisms. These reactions are catalyzed by tightly regulated enzymes, which sense environmental cues and provide energy, reducing power and macromolecules to supply the cellular needs. Metabolic reactions can be classified into catabolic pathways that produce energy (adenosine triphosphate, ATP) through the breakdown of molecules, and anabolic pathways that synthesize molecules through energy-consuming processes. The metabolic network is regulated by signaling pathways that respond to the specific cellular needs which, in turn, may vary depending on the cell type and proliferative state.

**Figure 1:**
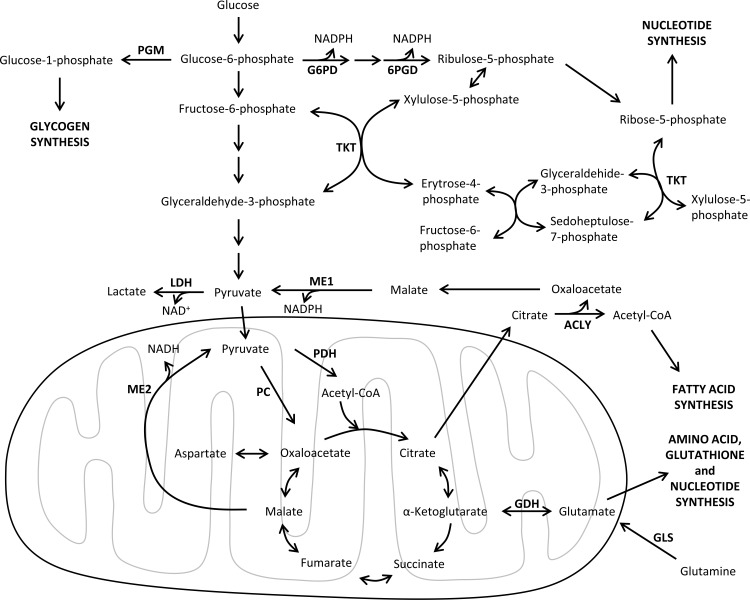
Major metabolic pathways involved in tumor metabolic reprogramming An overview of the main catabolic and anabolic metabolic pathways supporting tumor cell growth and survival. Enzymes are shown in bold. 6PGD, 6-phosphogluconate dehydrogenase; ACLY, ATP citrate lyase; CoA, coenzyme A; GLS, glutaminase; GDH, glutamate dehydrogenase; G6PD, glucose-6-phosphate dehydrogenase; GAPDH, glyceraldehyde-3-phosphate dehydrogenase; LDH, lactate dehydrogenase; ME1, malic enzyme 1 cytoplasmic form; ME2, malic enzyme 2 mitochondrial form; NAD^+^, nicotinamide adenine dinucleotide oxidized form; NADH, nicotinamide adenine dinucleotide reduced form; NADPH, nicotinamide adenine dinucleotide phosphate reduced form; PC, pyruvate carboxylase; PDH, pyruvate dehydrogenase; PGM, phosphoglucomutase.

Despite the fact that there are several metabolic similarities between tumor and highly proliferating non-transformed cells (reviewed in [18]), oncogenic regulation and tumor microenvironment have a distinctive influence on the metabolic reprogramming of cancer cells. In particular, tumor cells switch their core metabolism to meet the increased requirements of cell growth and division. Indeed, tumor metabolic reprogramming involves the enhancement of key metabolic pathways such as glycolysis, pentose phosphate pathway, glutaminolysis and lipid, nucleic acid and amino acid metabolism [19] (Figure 1). Thus, activation of oncogenic signaling pathways adapts tumor cells metabolism to the dynamic tumor microenvironment, where nutrient and oxygen concentrations are spatially and temporally heterogeneous [20, 21]. The dependencies on specific metabolic substrates such as glucose or glutamine exhibited by tumor cells are determined by the alterations in their oncogenes and tumor suppressor genes. For instance, MYC-transformed cells display addiction to glutamine as a bioenergetic substrate and are sensitive to inhibitors of glutaminolysis [22]. Accordingly, the characterization of the metabolic reprogramming of cancer cells and its connection with oncogenic signaling is a promising approach to identify novel molecular-targeted strategies in cancer therapy.

### Glycolysis and the Warburg effect

Glycolysis is the metabolic pathway by which glucose and other sugars are metabolized to pyruvate in an oxygen-independent manner to generate energy in the form of ATP and intermediates, which are used as precursors for the biosynthesis of macromolecules [23]. Under physiologic oxygen concentrations, pyruvate enters the mitochondria to be oxidized through an oxygen-dependent process known as oxidative phosphorylation (OXPHOS), which couples the oxidation of metabolites and the electron transport chain (ETC) with ATP production, being also a potential source of reactive oxygen species (ROS) [20].

The first metabolic phenotype observed in tumor cells was described by Otto Warburg as a shift from oxidative phosphorylation to aerobic glycolysis to generate lactate and ATP even in presence of oxygen, which is known as the Warburg effect [24, 25]. Therefore, cancer cells convert most incoming glucose to lactate rather than entering in the mitochondria to be oxidized through oxidative phosphorylation [26]. Initially, it was believed that the Warburg effect resulted from defects in the mitochondrial function of cancer cells. However, this effect is also exhibited by tumor cells with intact and functional mitochondria, suggesting that their preference for glycolysis might confer benefits on them such as reduced levels of ROS, high production of metabolic intermediates for macromolecular biosynthesis and acidification of extracellular microenvironment due to lactate excretion [27, 28]. It is worth noting that the ATP produced per molecule of glucose catabolized through glycolysis is considerably less efficient than through oxidative phosphorylation (2 versus 31-38 molecules of ATP [29], respectively), causing tumor cells to greatly increase both the rate of glucose uptake and glycolysis to sustain their increased energetic, biosynthetic and redox needs [30]. Conveniently, the high glycolytic rates displayed by cancer cells allow their visualization by ^18^F-deoxyglucose positron emission tomography (FDG-PET) and assist tumor detection, prevention and treatment [31].

Over the past decade, numerous studies and reviews have supported the hypothesis that the Warburg effect can be explained by the alterations in multiple signaling pathways resulting from mutations in oncogenes and tumor suppressor genes [21, 28, 32–35]. The complex network of mechanisms leading to the Warburg phenomenon includes mitochondrial changes, upregulation of rate-limiting enzymes in glycolysis involving specific isoforms such as M2 pyruvate kinase and hexokinase 2, intracellular pH regulation, and hypoxia-induced switch to anaerobic metabolism (reviewed in [35]). The enhanced glycolytic rate can be sustained through the overexpression of glucose transporters [36] and several key glycolytic enzymes [37] mediated by specific activated oncogenes (e.g. *PI3K* and *MYC*) and transcription factors (e.g. HIF1), contributing to the acquisition of the Warburg effect and maintaining tumor cell growth and survival [21, 28, 33]. Likewise, loss-of-function mutations in tumor suppressor *TP53* (encoding p53) also contribute to the Warburg effect, since they prevent i) p53-mediated transcriptional repression of glucose transporters GLUT1 and GLUT4; ii) activation of cytochrome c oxidase assembly protein (SCO2) expression, which promotes OXPHOS; and iii) upregulation of *TP53*-induced glycolysis and apoptosis regulator (TIGAR) expression, which reduces the intracellular concentration of the glycolytic activator fructose-2,6-bisphosphate [20, 38].

Interestingly, the metabolic switch in tumor cells has a key role in the establishment of many other cancer hallmarks [19]. In fact, some metabolic enzymes have been described as multifaceted proteins which can directly regulate transcription, glucose homeostasis and resistance to cell death [39, 40]. For example, hexokinase 2 isoform (HK2), which catalyzes the rate-limiting first step of glycolysis, plays a key role for the Warburg effect in cancer [41–43]. Specifically, HK2 bounds to mitochondria and is recognized as a signaling component controlling cellular growth, preventing mitochondrial apoptosis and enhancing autophagy [44, 45]. The competitive binding of HK2 to the voltage-dependent anion channel (VDAC) in the outer mitochondrial membrane prevents the union of VDAC with pro-apoptotic Bax, inhibiting cytochrome c release from mitochondria and avoiding apoptosis after Bax activation [44]. Therefore, targeting multifunctional metabolic enzymes may restore the susceptibility of tumor cells to cell death, offering new options for cancer therapy.

### Pentose phosphate pathway

Pentose phosphate pathway (PPP) is one of the main metabolic pathways that enables tumor cell proliferation by regulating the flux of carbons between nucleic acid synthesis and lipogenesis to support DNA replication and RNA production. DNA and RNA nucleic acids are polymers composed by combinations of four different nucleotides which in turn are constituted by an organic base (purine, in the case of the nucleotides adenine and guanine, or pyrimidine, in the case of cytosine, thymine, and uracil), a pentose sugar (ribose for RNA or deoxyribose for DNA) and one or more phosphate groups. The pentose phosphate is mainly obtained through the PPP, which also generates nicotinamide adenine dinucleotide phosphate (NADPH). NADPH is an essential cofactor for providing reducing equivalents for lipid and amino acid biosynthesis, and for modulating oxidative stress through the maintenance of the reduced glutathione (GSH) pool [46]. The association between upregulation of PPP and tumor cell proliferation is been extensively studied, as PPP plays a pivotal role in allowing tumor cells to meet their anabolic demands and counteract oxidative stress [47–49].

PPP is divided into the oxidative branch and the non-oxidative branch. The oxidative branch catalyzes the irreversible transformation of glucose-6-phosphate into ribose-5-phosphate (R5P), yielding NADPH. The non-oxidative branch is a reversible pathway that interconverts R5P and glycolytic intermediaries. The enzymes that mainly regulate the PPP are glucose-6-phosphate dehydrogenase (G6PD) in the oxidative branch and transketolase (TKT) in the non-oxidative branch [50–52]. Several oncogenic signaling pathways promote G6PD activation by post-translational mechanisms [46], while tumor suppressor p53 directly inhibits G6PD and the PPP [48]. PPP is coordinated with cell cycle since proliferating cells increase G6PD activity during late G1 and S phases [53]. Moreover, the activation of the SCF ubiquitin ligase by its interaction with the protein-b-transduction repeat-containing protein (b-TrCP) allows the recognition of PFKFB3 and its proteasome degradation during S phase [54, 55], promoting the shuttling of glycolytic substrates through the PPP and increasing the production of NADPH and R5P to allow S phase progression.

### Lipid metabolism

Triacylglycerides, phosphoglycerides, sterols and sphingolipids are hydrophobic or amphipathic molecules known as lipids. Fatty acids are long hydrocarbon chains with a carboxy-terminal group that constitute the main component of triacylglycerides and phosphoglycerides, being also present in sphingolipids and sterol esters. While triacylglycerides are used as energy storage units, phosphoglycerides, sterols and sphingolipids are major structural components of plasma membranes. Lipids are also involved in signal transduction and participate in the regulation of cell growth, proliferation, differentiation, survival, apoptosis, membrane homeostasis, motility and drug resistance [56, 57].

Tumor metabolic reprogramming involves an increase in lipid biosynthesis to supply the building blocks for membrane formation and sustain the high proliferative rate of tumor cells. Distinctively, tumor cells mainly activate and thrive on *de novo* lipid biosynthesis, while most non-transformed cells rely on extracellular lipids. Oncogenic signaling enhances lipogenesis through the increase of precursors for fatty acids synthesis (i.e. promoting glucose and glutamine transport, glycolysis, PPP and anaplerosis) and the upregulation of many lipogenic enzymes such as ATP citrate lyase (ACLY), fatty acid synthase (FASN) and acetyl-CoA carboxylase (ACC) [58–61]. The acetyl groups for fatty acids biosynthesis are provided by mitochondrial citrate, which is exported to the cytosol where ACLY catalyzes its conversion into acetyl-CoA and oxaloacetate [62]. Then, malate dehydrogenase (MDH) and malic enzyme (ME) can produce pyruvate from oxaloacetate, yielding part of the NADPH required for fatty acid biosynthesis. In addition, lipid biosynthesis is also connected to other pathways that generate NADPH, such as the oxidative branch of the PPP. Next, acetyl-CoA is converted to malonyl-CoA by ACC, and both acetyl and malonyl groups are condensed through a cyclical series of reactions by FASN, resulting in long-chain saturated fatty acids, predominantly palmitate. Further elongation and desaturation of *de novo* synthesized saturated fatty acids can be obtained through the action of elongases and desaturases [56, 63]. On the other hand, the mitochondrial degradation of fatty acids through β-oxidation releases large amounts of ATP and generates ROS through the TCA cycle and the oxidative phosphorylation [56, 57].

Sterol regulatory element-binding proteins (SREBPs) transcription factors regulate the expression of most enzymes involved in the synthesis of fatty acids and cholesterol. In turn, SREBPs are negatively regulated by tumor suppressors such as p53, pRB and AMPK, and activated by oncogenes such as PI3K and Akt. For instance, besides promoting glycolysis, Akt upregulates the expression of the lipogenic enzymes through activation and nuclear translocation of SREBP [64], and positively regulates ACLY by direct phosphorylation [65], linking enhanced glycolysis with increased lipogenesis [63, 66]. Therefore, targeting lipogenic pathways is thought to be a promising strategy for cancer therapy, as lipogenic enzymes are found to be upregulated or activated in tumor cells to satisfy their increased demand for lipids [57, 58].

### Amino acid metabolism

Amino acids are organic compounds containing a specific side chain and both amino and carboxyl groups that enable them to undergo polymerization to form proteins. In addition, amino acids can be metabolized as a source of carbon and nitrogen for biosynthesis. There are 20 different amino acids, 11 of which can be endogenously synthesized by mammal cells while the remainder are known as essential amino acids, which must be obtained from external sources. In fact, amino acids have a pivotal role in supporting proliferative metabolism and are required for cell survival. It is not surprising that cells have developed an amino acid sensing system through the mechanistic target of rapamycin (mTOR) signaling to determine whether there are sufficient amino acids available for protein biosynthesis. Specifically, leucine, glutamine and arginine serve as critical signaling molecules that activate mTOR pathway [67, 68]. In response to amino acid deficiency, inhibition of mTOR rapidly suppress protein synthesis and induce autophagy, in order to maintain a free amino acid pool which may be required during prolonged amino acid limitation [69].

Non-essential aminoacids can be synthesized from glycolytic intermediates such as 3-phosphoglycerate, which is the precursor for serine, or pyruvate, that can be converted to alanine. In addition, TCA intermediates like oxaloacetate and α-ketoglutarate can generate aspartate, asparagine and glutamate. Moreover, glutamate can be converted to L-glutamate-5-semialdehyde (GSA) and 1-pyrroline-5-carboxylate (P5C), which are further converted to ornithine and proline, respectively [70]. Then, ornithine can enter the urea cycle and produce arginine. Also, serine can generate glycine and contribute to the synthesis of cysteine [71].

Highly proliferating cells, like tumor cells, consume essential and non-essential amino acids from external sources since the capacity of endogenous synthesis is not sufficient to fulfill their amino acidic increased needs [72]. However, most amino acids are hydrophilic molecules that require selective transport proteins to cross the cell membrane. Accordingly, four amino acid transporters (SLC1A5 [22, 73], SLC7A5 [73], SLC7A11 [74] and SLC6A14 [75]) have been found to be overexpressed in cancer cells in a MYC-dependent manner or through miR-23a repression mediated by MYC to increase the uptake of amino acids and meet their growing demands [72]. Interestingly, the functional coupling of SLC1A5 and SLC7A5 glutamine transporters suggests that enhanced glutamine metabolism in tumor cells can contribute to drive tumor growth through activation of mTOR [68].

In tumor cells, the consumption of some amino acids (specially non-essential amino acids) greatly exceeds the requirements for protein biosynthesis, suggesting their use as intermediates in metabolism by providing one carbon units, replenishing the TCA cycle or synthesizing fatty acids, nucleotides and other amino acids [71]. For example, glutamine, glycine and aspartate are required for nucleotide biosynthesis, while serine and glycine play an essential role in a one-carbon metabolism, generating precursors for the biosynthesis of lipids, nucleotides and proteins, regulating the redox status and participating in protein and nucleic acid methylation [76, 77]. The conversion of serine to glycine can be catalyzed either by the cytosolic or mitochondrial serine hydroxymethyltransferase (SHMT1 and SHMT2, respectively). Interestingly, the metabolic activity of SHMT2 has been shown to strongly correlate with the rates of proliferation across the NCI60 cancer cell collection [78]. In fact, SHMT2 has been suggested as fundamental to sustain cancer metabolism by fuelling heme biosynthesis and thus oxidative phosphorylation [79].

It is worth noting that the reactions catalyzing the degradation of proline produce significant amounts of ROS. The first step of proline degradation is catalyzed by the mitochondrial proline dehydrogenase (PRODH), which is a tumor suppressor that inhibits proliferation and induces apoptosis [70, 80]. This mitochondrial enzyme is linked to the electron transport chain through complex III, being shown as a source of ROS generation. In addition, P5C and proline can act as a redox couple, carrying reducing potential into and oxidizing potential out of the mitochondria by the combined activities of mitochondrial PRODH and the cytosolic form of P5C reductase (PYCR), which preferably uses NADPH [70, 80, 81].

It is worth noting that glutamine is the amino acid presenting the most prominent role in tumor metabolism. Accordingly, some tumor cells have been reported to exhibit dependence on glutamine for survival [22, 82].

### Mitochondrial metabolism

Mitochondrial function is essential for cancer cells as it is involved in numerous crucial cellular processes such as ATP generation, regulation of programmed cell death, and regulation of signal transduction pathways through ROS production, modulation of cytosolic calcium levels and trafficking of small metabolites. Indeed, impairment of mitochondrial function and reduction of mitochondrial biogenesis greatly suppresses tumor formation, growth and proliferation [63, 83, 84]. Conversely, enhancement of mitochondrial biogenesis is advantageous for tumor cells [63, 85]. On the other hand, alterations in mitochondrial function can lead to several diseases including cardiovascular dysfunctions, muscular degeneration and cancer [83, 86].

**Figure 2:**
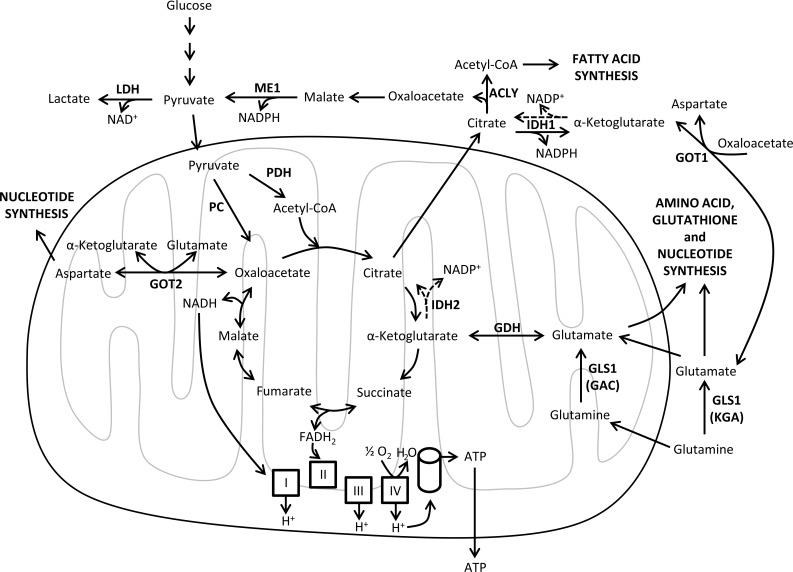
Mitochondrial metabolism Schematic representation of the biosynthetic and bioenergetic reactions of the TCA cycle and the oxidative phosphorylation (OXPHOS). OXPHOS is coupled to the oxidation of NADH and FADH_2_, which are produced in the TCA cycle, via the electron transport chain (ETC, also known as the mitochondrial respiratory chain). The ETC comprises four complexes (I to IV) that transfer electrons generating a gradient of protons (H^+^) in the mitochondrial intermembrane space, which is used by the ATPase (complex V) to produce ATP. Reductive carboxylation of α-ketoglutarate by IDH1 and IDH2 produces citrate (dashed arrows). ACLY, ATP citrate lyase; ATP, adenosine triphosphate; CoA, coenzyme A; FADH_2_, flavin adenine dinucleotide reduced form; GAC, glutaminase C; GDH, glutamate dehydrogenase; GLS1, glutaminase 1; GOT1, glutamic-oxaloacetic transaminase 1 cytoplasmic form; GOT2, glutamic-oxaloacetic transaminase 2 mitochondrial form; IDH1, isocitrate dehydrogenase cytoplasmic form; IDH2, isocitrate dehydrogenase mitochondrial form; KGA, kidney (K-type) glutaminase; LDH, lactate dehydrogenase; ME1, malic enzyme 1 cytoplasmic form ; NAD^+^, nicotinamide adenine dinucleotide oxidized form; NADH, nicotinamide adenine dinucleotide reduced form; NADP^+^, nicotinamide adenine dinucleotide phosphate oxidized form; NADPH, nicotinamide adenine dinucleotide phosphate reduced form; PC, pyruvate carboxylase; PDH, pyruvate dehydrogenase.

In the presence of oxygen, oxidative phosphorylation (OXPHOS) is the most efficient mechanism for synthesizing ATP [29]. OXPHOS is coupled to the oxidation of reduced nicotinamide adenine dinucleotide (NADH) and flavin adenine dinucleotide (FADH_2_) through the electron transport chain. The mitochondrial respiratory chain, located in the mitochondrial inner membrane, comprises four complexes (I to IV) that are responsible for the oxidation of the reducing equivalents in the form of NADH or FADH_2_ and the reduction of molecular oxygen (final electron acceptor) to water. This process is coupled to the pumping of protons into the mitochondrial intermembrane space, resulting in a proton gradient that is used by the ATPase (complex V) to produce ATP [87] (Figure 2).

Among the metabolic pathways that take place in the mitochondria, the tricarboxylic acid (TCA) cycle is a route of pivotal importance for the entire cellular metabolism and, in particular, for oxidative metabolism. Remarkably, TCA cycle provides precursors for the biosynthesis of lipids, nucleic acids and proteins, as well as reducing equivalents (NADH and FADH_2_) for ATP production (Figure 2). Mutations in several genes that encode enzymes of the TCA cycle including isocitrate dehydrogenase [88, 89], succinate dehydrogenase and fumarate hydratase [90] are associated with some tumor types, leading to the dysfunction of the TCA cycle and the accumulation of its substrates [86, 91, 92]. Interestingly, it has been shown that increased levels of TCA cycle intermediates fumarate and succinate can affect α-ketoglutarate-dependent histone and DNA demethylases, HIF stabilization, and cellular responses to O_2_ depletion [90, 93]. Isocitrate dehydrogenases (IDHs) catalyze the oxidative decarboxylation of isocitrate to α-ketoglutarate, which is required as a substrate for numerous dioxygenases, including histone demethylases, prolyl hydroxylases, collagen prolyl-4-hydroxylases, and the TET family of 5-methylcytosine hydroxylases [94, 95]. There are three IDH enzymes; IDH1 is found in the cytosol and peroxisome, while IDH2 and IDH3 isoforms are localized in the mitochondria. Mutations targeting IDH1 and IDH2 result in loss of their native enzymatic activities and lead to the production of 2-hydroxyglutarate, a metabolite that can competitively inhibit α-ketoglutarate-dependent dioxygenases and is associated with tumorigenesis [95–97].

#### Glutamine metabolism

Glutamine is the most abundant amino acid in plasma and in intracellular pools, being consumed at significantly higher rates than other amino acids by tumor cells [82]. Glutamine plays several cellular key roles as a nitrogen donor for nucleotide and protein synthesis, as a carbon source for energy production and lipid biosynthesis, and as a precursor for some non-essential amino acids and the antioxidant GSH biosynthesis [82, 98]. Despite being a non-essential amino acid, glutamine is crucial for the proliferation of most cells and for the viability of some tumor cells that have developed glutamine dependence [99].

The expression levels of oncogenes (e.g. *MYC* [22, 100], *mTOR* [101] and *KRAS* [102]) and tumor suppressors (e.g. *SIRT4* [103] and *TP53* [104]) are decisive to regulate glutamine metabolism [92, 105], to the extent that tumor genetics can dictate cellular dependence on glutamine for survival [98]. For instance, tumor cells overexpressing MYC reprogram their mitochondrial metabolism to depend on glutamine for the maintenance of cell viability, mitochondrial integrity and TCA cycle anaplerosis, triggering cellular addiction to glutamine and displaying increased sensitivity to glutamine deprivation [22, 100].

In addition to glycolysis, many tumor cells also rely on glutamine to fulfill their bioenergetic and metabolic needs. Indeed, glutamine catabolism is the source of many precursors for major anaplerotic processes such as the TCA cycle. Cells requiring *de novo* lipid biosynthesis, like tumor cells, divert citrate from the TCA cycle to produce lipogenic acetyl-CoA. The depletion of citrate from the TCA cycle creates a need for anaplerotic replenishment of the cycle, which can be provided through oxidative metabolism of glutamine [106]. Oxidation of glutamine in the mitochondria begins with its conversion to glutamate catalyzed by glutaminase (GLS). Glutaminase is a pivotal enzyme in the regulation of glutamine metabolism in tumor cells which has recently gathered some attention as a promising target for cancer therapy [107–109]. There are three mammalian glutaminase isoforms; kidney (K-type) glutaminase (KGA) and glutaminase C (GAC) are encoded by *GLS* and referred to as GLS1, while liver (L-type) glutaminase (LGA) is encoded by *GLS2* and usually known as GLS2 [110]. Glutamate can be converted to α-ketoglutarate by either glutamate dehydrogenase (GDH) or transaminases, to feed the TCA cycle (Figure 2). In addition, glutamate can serve as a precursor of GSH and non-essential amino acids such as aspartate, alanine, proline and arginine. Interestingly, α-ketoglutarate levels are determinant for the regulation of HIF1α degradation through prolyl hydroxylase (PHD) sensing pathway [67, 94]. Furthermore, glutaminolysis and α-ketoglutarate are also involved in the activation of mTOR signaling [68, 111]. Glutamine carbons can exit the TCA cycle in the form of malate, which can be converted to pyruvate by malic enzyme (ME) with NADPH generation [112]. Both glutamine-derived NADPH and GSH production allow tumor cells to reduce the oxidative stress associated with mitochondrial respiration and rapid cell proliferation.

It is worth noting that glutamine utilization as a respiratory substrate through the TCA cycle produces NADH and FADH_2_ that provide electrons for the mitochondrial electron transport chain to generate ATP (Figure 2). Remarkably, glycolytic contribution to total ATP synthesis in tumor cells differs widely depending on cell type, from over 60% to less than 1%, with a mean contribution of 17±18% in the tested cell lines [113]. These results are confirmed by a flux balance analysis across the NCI-60 cell lines [78, 114] which shows that oxidative phosphorylation contributes to 70-84% of the total cellular ATP production [115]. Therefore, oxidative metabolism of glutamine is the major energetic source in many cancer cell lines.

Together, glucose and glutamine are the two principal nutrients to coordinately fuel the proliferation of tumor cells by supplying not only ATP but also key precursors for protein, lipid and nucleic acid biosynthesis (Figures 1 and 2). In fact, some cancer cells can switch their carbon source in response to nutrient availability. For example, glucose withdrawal increases GDH activity in MYC-transformed glioblastoma cells [116], while impairing the oxidative metabolism of glutamine by silencing glutaminase induces a compensatory anaplerotic mechanism catalyzed by pyruvate carboxylase (PC) that enables the use of glucose-derived pyruvate for anaplerosis [117]. However, the metabolic compensation adopted by tumor cells renders them absolutely dependent on the new upregulated pathways, opening new opportunities for cancer combined therapies. Therefore, the metabolic flexibility and compensatory abilities exhibited by some tumor cells have to be carefully considered when designing cancer therapies.

#### Glutamine reductive carboxylation

There are two different glutamine-dependent pathways for fatty acid biosynthesis. On the one hand, cells can oxidatively metabolize glutamine-derived α-ketoglutarate to citrate in the TCA cycle and subsequently transport it to the cytosol to generate oxaloacetate and lipogenic acetyl-CoA [62]. Likewise, malate produced from glutamine in the TCA cycle can generate pyruvate through the action of malic enzyme, which can be further metabolized to lipogenic acetyl-CoA. On the other hand, α-ketoglutarate obtained from glutamine can be directly converted to citrate by reductive carboxylation, especially in tumor cells under hypoxic conditions or when mitochondrial respiration is impaired, in order to sustain cell growth under these circumstances [118–121]. This reaction takes advantage of the reversible transformation catalyzed by aconitase and isocitrate dehydrogenase. The cytosolic NADP^+^/NADPH-dependent isocitrate dehydrogenase 1 (IDH1) is the main enzyme catalyzing the reversible reductive carboxylation of α-ketoglutarate to isocitrate and NADP^+^ [118] (Figure 2). Indeed, reductive carboxylation of glutamine provides a glucose-independent pathway to generate acetyl-CoA for biosynthesis, allowing cells to conserve glucose for the production of biosynthetic precursors that are specifically generated from glucose [118].

## ONCOGENIC REGULATION OF TUMOR METABOLIC REPROGRAMMING

Tumor metabolic reprogramming is a direct result of the re-engineering of intracellular signaling pathways that are altered by mutations in oncogenes and tumor suppressor genes. In fact, most cancers harbor activating mutations of oncogenes and/or inactivating mutations of tumor suppressor genes which determine the tumor metabolic phenotype and support tumorigenesis by giving to transformed cells a proliferative advantage over non-malignant cells. Several oncogenes including MYC, hypoxia inducible factor 1 (HIF1), phosphoinositide-3-kinase (PI3K), protein kinase B (PBK or Akt) and the mechanistic target of rapamycin (mTOR), have been known to be involved in the regulation of tumor metabolic reprogramming [5, 20, 92].

### MYC as a master regulator of tumorigenesis

The *MYC* oncogene belongs to the *MYC* family of genes together with *MYCN* and *MYCL*. However, *MYC* is the only isoform ubiquitously expressed in a broad range of tissues, while *MYCN* and *MYCL* are normally only expressed during development [122]. MYC is a multi-functional transcription factor that exerts control over cell proliferation, cell cycle progression, cell growth, metabolism, apoptosis, differentiation and stress response through transcriptional regulation of its target genes [122, 123]. In fact, MYC binds to the promoter of 10-15% of all known genes, regulating both genes encoding proteins and those encoding non-coding RNA products of several functional classes [122, 124]. *MYC* expression is dysregulated in many human cancers by either chromosomal translocation or gene amplification. In addition, the expression and stability of MYC protein and *MYC* mRNA can also be dysregulated, promoting tumorigenesis through unrestricted cell proliferation, inhibition of cell differentiation, metabolic adaptation, angiogenesis, reduction of cell adhesion and genomic instability [122, 123, 125, 126].

To function as a transcription factor, MYC protein heterodimerizes with its binding partner MAX, forming an activated complex that recognizes E box sequences (CACGTG) and induces the transcription of its targets genes. MYC can also act as transcriptional repressor by binding to MIZ1 or SP1 transcription factors and interfering with their transcriptional activity [127]. It is worth noting that multiple genes that are repressed by MYC encode negative regulators of cell proliferation such as *CDKN2B* (encoding p15^INK4b^), *CDKN2C* (p18^INK4c^), *CDKN1A* (p21^Cip1^), *CDKN1B* (p27^Kip1^), and *CDKN1C* (p57^Kip2^) [127]. MAX can also bind to MAD1, MXI1, MAD3, MAD4 and MNT or form homodimers, repressing the transcriptional activation of MYC target genes [128].

#### MYC and metabolism

**Figure 3:**
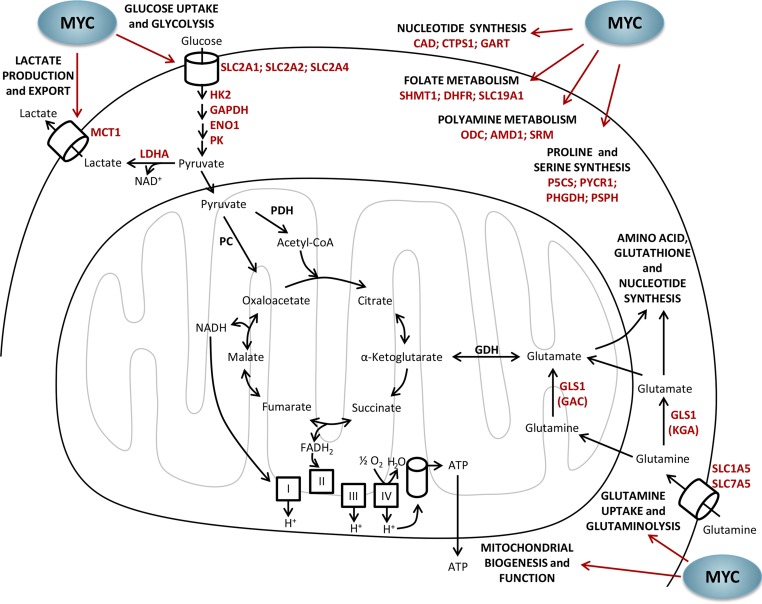
Metabolic regulation by MYC MYC has a pivotal role in the metabolic reprogramming of tumor cells by enhancing glucose uptake and glycolysis, lactate production and export, glutamine uptake and glutaminolysis, mitochondrial biogenesis and oxidative phosphorylation, and nucleotide, folate, polyamine, proline and serine synthesis. AMD1, adenosylmethionine decarboxylase; ATP, adenosine triphosphate; CAD, carbamoyl-phosphate synthase/aspartate carbamoyltransferase/dihydroorotase; CoA, coenzyme A; CTPS1, cytidine triphosphate synthase 1; DHFR, dihydrofolate reductase; FADH_2_, flavin adenine dinucleotide reduced form; GAC, glutaminase C; GART, phosphoribosylglycinamide formyltransferase, phosphoribosylglycinamide synthetase, phosphoribosylaminoimida-zole synthetase; GDH, glutamate dehydrogenase; GLS1, glutaminase 1; KGA, kidney (K-type) glutaminase; LDHA, lactate dehydrogenase A; MCT1, monocarboxylate transporter/SLC16A1 solute carrier family 16 member 1; NAD^+^, nicotinamide adenine dinucleotide oxidized form; NADH, nicotinamide adenine dinucleotide reduced form; NADP^+^, nicotinamide adenine dinucleotide phosphate oxidized form; NADPH, nicotinamide adenine dinucleotide phosphate reduced form; ODC, ornithine decarboxylase; P5CS, Δ^1^-pyrroline-5-carboxylate synthetase; PHGDH, phosphoglycerate dehydrogenase; PC, pyruvate carboxylase; PDH, pyruvate dehydrogenase; PSPH, phosphoserine phosphatase; PYCR1, Δ^1^-pyrroline-5-carboxylate reductase 1; SHMT1, serine hydroxymethyltransferase 1; SLC1A5, solute carrier family 1 (neutral amino acid transporter) member 5; SLC2A1, solute carrier family 2 (facilitated glucose transporter) member 1; SLC2A2, solute carrier family 2 (facilitated glucose transporter) member 2; SLC2A4, solute carrier family 2 (facilitated glucose transporter) member 4; SLC7A5, solute carrier family 7 (amino acid transporter light chain, L system) member 5; SLC19A1, solute carrier family 19 (folate transporter) member 1; SRM, spermidine synthase.

MYC is known to enhance glycolysis through the activation of glycolytic genes (such as *HK2*, *GAPDH*, *ENO1* and *PK*, among others) and glucose transporters (*SLC2A1*, *SLC2A2* and *SLC2A4*) [129, 130]. In addition, MYC promotes lactate production and export, increasing the gene expression of *LDHA* and lactate transporter *MCT1* [126, 131, 132]. Figure 3 illustrates the main metabolic pathways regulated by MYC.

On the other hand, transformed cells exhibit increased MYC-dependent glutaminolysis and glutamine dependency [22, 73]. Indeed, MYC has been described as the main oncoprotein responsible for inducing a transcriptional program that promotes glutaminolysis and triggers cellular addiction to glutamine as a bioenergetic substrate [22]. This glutamine addiction leads tumor cells to reprogram intermediate metabolism for the maintenance of mitochondrial tricarboxylic acid (TCA) cycle integrity [22]. Moreover, high levels of MYC promote mitochondrial biogenesis and function, both increasing the rate of oxygen consumption and the energy production required for rapid cell proliferation [125, 133–135]. High glutaminolysis rate results in the robust production of NADPH, which is needed to fulfill the requirements for cell proliferation [22, 112]. In conditions of low glucose and oxygen availability, MYC-induced glutamine catabolism is important for cell survival [108]. Furthermore, cells with supraphysiological levels of MYC are more sensitive to inhibition of mitochondrial oxidative metabolism [136]. Moreover, MYC is also found to contribute to increase glutamine uptake by upregulation of the expression of glutamine transporters (ASCT2 [SLC1A5] and SLC7A5) [73, 85]. Importantly, MYC enhances glutaminolysis by transcriptionally repressing miR-23a and miR-23b (microRNA-23a/b), resulting in greater expression of their target protein, glutaminase (GLS1) [73]. In fact, GLS1 is the first enzyme in the glutaminolysis and catalyzes the conversion of glutamine to glutamate for its oxidation in the TCA cycle and also for protein or glutathione synthesis. It is worth mentioning that MYC can stimulate the use of the TCA cycle to generate intermediates for macromolecular synthesis using both glucose and glutamine as carbon source [98, 108]. However, it has been reported that cells presenting high MYC levels greatly rely on mitochondrial oxidative phosphorylation and increase glutaminolysis by 2- to 4-fold, while only moderately increasing glycolysis by 1.2-fold [137].

Additionally, MYC has been shown to activate nucleotide biosynthesis by inducing several gens involved in nucleotide metabolism including *carbamoyl-phosphate synthase / aspartate carbamoyltransferase / dihydroorotase* (*CAD*), *CTP synthase 1* (*CTPS*) and *ornithine decarboxylase* (*ODC*) [129, 132, 138, 139]. Polyamine biosynthesis is also regulated by MYC since ornithine decarboxylase (ODC) (the rate-limiting enzyme in polyamine production) [138], adenosylmethionine decarboxylase (AMD1) and spermidine synthase (SRM) have E boxes in their regulatory region and are enhanced in MYC-expressing cells [132]. Furthermore, polyamines stimulate MYC transcription in a positive feedback loop [140, 141].

Moreover, MYC can redirect glycolytic flux from 3-phosphoglycerate for the synthesis of serine and glycine involving folate metabolism, which are essential for purine and thymidylate biosynthesis [85, 132, 142, 143]. MYC is also implicated in proline metabolism regulation by transcriptionally repressing proline oxidase/proline dehydrogenase (POX/PRODH) expression through upregulation of miR-23b*, and increasing the expression of the enzymes of proline biosynthesis pathway (P5C synthase, P5CS and P5C reductase 1, PYCR1) [70].

#### MYC and cell cycle

The network of MYC target genes suggests its implication in the fulfillment of the metabolic requirements for cell cycle entry [136, 144]. In fact, one of the earliest observations after *MYC* discovery was its ability to promote cell proliferation and inhibit cell differentiation [129]. Remarkably, MYC overexpression in quiescent cells is sufficient to trigger cell cycle entry, reduce the requirement for growth factors, block cell cycle exit, and increase cell size [145, 146]. MYC promotes cell cycle progression by regulation of pivotal cell cycle control genes through transcriptional induction of CDKs and cyclins, and repression of CIP/KIP proteins. MYC mediates the increase of active cyclin-CDK complexes levels not only by upregulation of *CDK1*, *CDK2*, *CDK4* (one of the principal MYC target genes [147]), *CDK6*, *CCND1* (encoding cyclin D1), *CCND2* (cyclin D2), *CCND3* (cyclin D3), *CCNE1* (cyclin E1), *CCNE2* (cyclin E2), *CCNA2* (cyclin A2) and *CCNB1* (cyclin B1), but also by induction of CDC25A (CDKs phosphatase) and CDK activating kinase complex (CAK, through enhanced mRNA translation of its subunits, CDK7, cyclin H and MAT1), and repression of the CDK inhibitory kinase *WEE1* through miR-221 activation [122, 126, 129, 145, 148]. Moreover, MYC abrogates the transcription of cell cycle checkpoint genes *GADD45* and *GADD153* [122, 148], and impairs the activity of the CDK inhibitors p27^Kip1^, p21^Cip1^ and p15^INK4b^ through several mechanisms [122, 126, 129, 145]. One of the most studied mechanisms for p21^Cip1^ and p15^INK4b^ MYC-mediated repression is the binding to MIZ1 and the blocking of its transcriptional activity [129, 145]. In contrast, MYC antagonizes p27^Kip1^ function by several parallel mechanisms such as induction of miR-221 and miR-222, activation of E2F transcription factors, increase of CDK4/6-cyclin D and CDK2-cyclin E complexes levels, and enhancement of the expression of several components of the SCF ubiquitin ligase complex [122, 145]. Last but not least, MYC further stimulates cell cycle progression by inducing genes directly involved in DNA replication including *MCM* (minichromosome maintenance), *ORC* (origin recognition complex), *CDC6* (cell division cycle 6), *TERT* (telomerase reverse transcriptase) and the genes encoding three subunits of the APC/C (*ANAPC5*, *CDC16* and *CDC23*) [145].

#### MYC regulation

Given its pivotal role on cell fate, *MYC* expression is tightly regulated at transcriptional, post-transcriptional and post-translational levels in non-transformed cells. Accordingly, dysregulation of *MYC* expression is one of the most common abnormalities in human diseases, being MYC overexpression frequently found in most human cancers. Remarkably, *MYC* oncogenic activation results from insertional mutagenesis, chromosomal translocation and gene amplification mechanisms, while most oncogenes are activated by mutations in their coding sequence [122].

MYC protein presents extremely short half-life (in the order of 20-30 minutes [149]) in the absence of mitogenic signals, but is transiently stabilized upon cell cycle entry and RAS activation, allowing its accumulation [150, 151]. RAS promotes MYC stability through RAF/MEK/ERK kinase cascade and via glycogen synthase kinase-3β (GSK-3β) inhibition by the PI3K/Akt pathway [150, 152]. MYC turnover is regulated by the ubiquitin proteasome pathway [149, 153] and is dependent on the phosphorylation of two highly conserved residues located near the N-terminal region of *MYC*, Thr58 and Ser62. These phosphorylation sites exert opposing control effects on MYC degradation [150]. ERK (extracellular receptor kinase) phosphorylates MYC on Ser62, promoting its protein accumulation, while phosphorylation of Thr58, which is mediated by GSK-3β but dependent on prior Ser62 phosphorylation, triggers MYC proteasomal degradation [150–152, 154]. Therefore, MYC phosphorylation at Ser62 has two opposite roles; MYC stabilization and accumulation, and activation of the subsequent phosphorylation at Thr 58, triggering MYC degradation. Interestingly, proteasome inhibition studies reveal that the accumulated poly-ubiquitinated MYC only exhibits phosphorylation on Thr58 [152, 154]. Since phosphorylation on Ser62 is required prior to Thr58 phosphorylation, the Ser62 phosphate is removed before MYC ubiquitination by protein phosphatase 2A (PP2A) action, contributing to MYC degradation [150, 152, 154].

In non-transformed cells, growth stimuli lead to RAS activation and MYC protein synthesis. However, when mitogenic signaling ends, RAS and PI3K activities decline and release GSK-3β from its negative regulation, activating its kinase activity and thus promoting MYC degradation by phosphorylation on Thr58 [154]. The ordered phosphorylation of Ser62 and Thr58 followed by Ser62 dephosphorylation allows a tight control of MYC protein levels. Hence, the disruption of the physiological regulation of MYC expression can lead to malignancy.

### HIF1

**Figure 4:**
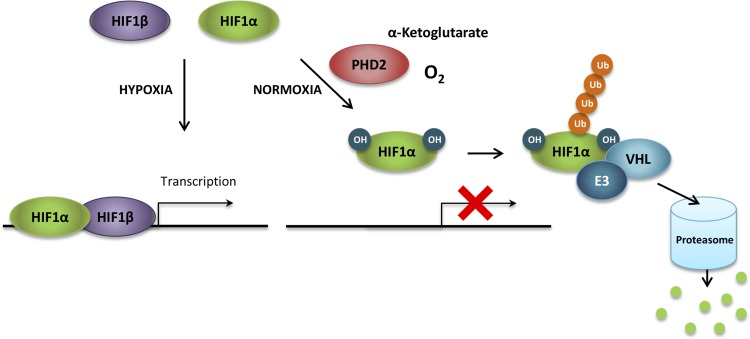
HIF1α regulation under normoxia In the presence of oxygen and α-ketoglutarate, HIF1α subunits are hydroxylated on proline residues in the oxygen-dependent degradation (ODD) domain by prolyl hydroxylases (principally prolyl hydroxylase 2, PHD2). Prolyl hydroxylation is required for the binding of the von Hippel-Lindau protein (VHL), which recruits an ubiquitin ligase complex (E3) that ubiquitinates HIF1α. Ubiquitination marks HIF1α for proteasomal-mediated degradation. HIF, hypoxia inducible factor; OH, hydroxylation; Ub, ubiquitin.

The hypoxia inducible factors HIF1, HIF2 and HIF3 are the principal regulators of the transcriptional homeostatic responses to situations of limited availability of oxygen. HIF1 is ubiquitously expressed while HIF2 and HIF3 are only expressed in certain tissues [155]. Only HIF1 and HIF2 are further discussed in this section since HIF3 function is less well understood. The HIF factors are composed of an oxygen-dependent HIFα subunit and a constitutively expressed HIFβ subunit. HIF activity is tightly regulated by cycles of synthesis and oxygen-dependent proteasomal degradation. Indeed, HIFα subunits are continuously synthesized and their stability is regulated by oxygen availability [155]. Under normoxic conditions, HIFα subunits are hydroxylated on proline residues in the oxygen-dependent degradation (ODD) domain by prolyl hydroxylase enzymes (PHDs) and subsequently ubiquitinated by the tumor suppressor protein von Hippel-Lindau (VHL) prior to their degradation in the proteasome [155, 156] (Figure 4). Under hypoxic conditions, the reduced molecular oxygen levels decrease the activity of PHDs, which are further inactivated through the oxidation of the ferrous ion within their active sites by ROS released from inefficient mitochondrial respiration [157], thus preventing their interaction with VHL [156]. Consequently, stable HIFα subunits form heterodimers with HIFβ subunits and translocate to the nucleus, where they bind to specific consensus sequences (hypoxia response element, HRE) in the promoter of hypoxia-responsive genes for the transcriptional activation of the cellular adaptation to hypoxia [158].

#### Hypoxia and cancer

Solid tumors frequently develop hypoxia when highly proliferating tumor cells outgrow their vascular network, resulting in tumors with limited oxygen diffusion. In order to adapt to the hypoxic microenvironment and support cell survival, cells principally initiate response mechanisms through HIFα stabilization and accumulation, favoring angiogenesis, invasion and metabolic reprogramming [159, 160]. Accordingly, HIFα levels are increased in many human cancers and correlate with poor clinical prognosis [160]. It is worth mentioning that tumor cells can exhibit augmented levels of HIF1α under normoxic conditions, a phenomenon known as pseudo-hypoxia [159]. For example, induction of RAS or SRC oncogenic signaling promotes normoxic HIF1α accumulation through prolyl hydroxylation inhibition [161].

#### Regulation of HIF by prolyl hydroxylases

In humans, there are three different members of the prolyl hydroxylase family; PHD1, PHD2 and PHD3. However, only PHD2 has been confirmed to be involved in the oxygen regulation of HIF1α, while PHD1 and PHD3 display only partial additive effects on HIF1α stability [162]. These enzymes are good oxygen sensors since their affinity for oxygen is low with *K_m_* values from 230 to 250 μM, slightly above the concentration of oxygen in the air (200 μM) [163]. PHDs require α-ketoglutarate, oxygen and a prolyl residue as substrates, and iron and ascorbate as cofactors, to produce a hydroxyl-prolyl residue, succinate and CO_2_ [94]. Prolyl hydroxylation is required for the recognition and binding of VHL to the ODD domain, which recruits an ubiquitin ligase complex [158] (Figure 4). Chemical inhibitors of the activity of PHD, such as iron chelators (e.g. desferrioxamine, DFO) or competitors of α-ketoglutarate for binding at the hydroxylase (e.g. dimethyloxalylglycine, DMOG), prevent the hydroxylation of HIFα subunits, causing their accumulation and promoting the expression of HIF target genes [164]. Remarkably, the use of α-ketoglutarate as an electron donor in the reaction of hydroxylation results in its oxidation into succinate, which is an end product whose accumulation can inhibit PHD activity even in the presence of oxygen [93]. In fact, deficiency of succinate dehydrogenase has been demonstrated to increase succinate levels and competitively inhibit PHDs under normoxia, leading to HIF1α stabilization in a pseudo-hypoxic phenotype [93]. Interestingly, PHD activity can be rescued by artificially increasing cellular α-ketoglutarate levels both in normoxia, reversing the succinate-mediated HIF1α stabilization [165], and hypoxia, resulting in the destabilization of HIF1α and reversing the hypoxic phenotype [166]. Therefore, PHD activity is regulated not only by oxygen availability, but also by the availability of α-ketoglutarate, a metabolite which plays a central role in numerous metabolic processes and is closely connected to amino acid metabolism [94]. In fact, both intracellular α-ketoglutarate levels and PHD activity are highly dependent on amino acid availability, while amino acids ability to induce mTORC1 signaling requires PHD enzymatic activity [167].

It is worth noting that, in addition to the principal mechanism regulating HIF1α stability in response to oxygen availability involving PHD and VHL, there are also oxygen-independent pathways regulating the synthesis and degradation of HIF1α, which involve RACK1 (receptor for activated C kinase 1) protein binding to HIF1α, recruitment of an ubiquitin ligase complex and consequent HIF1α proteasome-mediated degradation [158, 168].

#### HIF transcriptional targets

HIF1α and HIF2α overlap in their ability to activate target genes involved in angiogenesis, metastasis and invasion, while HIF1α alone regulates several glycolytic and apoptotic genes and HIF2α preferentially promotes the transcription of certain genes such as *vascular endothelial growth factor* (*VEGF*) or *transforming growth factor α* (*TGFα*) [155, 159, 164, 169].

HIF1 activation increases oxygen and nutrients supply to tumors through angiogenesis and erythropoiesis stimulation by *VEGF* and *erythropoietin* (*EPO*) upregulation, respectively [21]. In addition, as part of the molecular mechanisms associated with the Warburg effect, HIF1 enhances glycolysis and lactate production by transactivating glucose transporters and glycolytic enzymes [170]. In accordance with the increased aerobic glycolysis, HIF1 prevents the mitochondrial oxidation of pyruvate, the final product of glycolysis, by inhibiting the activity of pyruvate dehydrogenase (PDH) through *pyruvate dehydrogenase kinase 1* (*PDHK1*) induction [171]. Furthermore, HIF1 enhances electron transport chain efficiency through *cytochrome c oxidase subunit IV, isoform 2* (*COX4I2*) induction, which replaces the less efficient isoform 1 (COX4I1), resulting in increased ATP production and reduced ROS generation [172]. In addition to the catabolic process of anaerobic glycolysis, HIF1 also endorses anabolic processes such as glycogen synthesis by upregulating the enzymes involved in its biosynthetic pathway [173–175].

#### HIF1 effects on MYC

There is a complex interplay between HIF1 and MYC proteins concerning glucose metabolism and mitochondrial function [131, 156, 176]. Both HIF1 and MYC share common metabolic target genes such as *SLC2A1* glucose transporter, *HK*, *phosphofructokinase* (*PFK*), *pyruvate kinase* (*PK*) or *LDH*, among others [131]. In contrast, *SLC2A3* glucose transporter is a specific HIF1 target gene [131]. On the other hand, HIF1 and MYC have opposing effects on cell proliferation, mitochondrial biogenesis and DNA repair [176]. HIF1 impairs mitochondrial biogenesis and oxygen consumption by inhibiting MYC-mediated transcription and inducing MYC degradation [134], while regulates cell cycle and DNA repair genes by functionally counteracting MYC through displacement of MYC inhibitory binding from the *CDKN1A* promoter [177] and of MYC activating binding from *MSH2* and *MSH6* promoters [178]. Remarkably, HIF1 directly inhibits MYC through induction of MXI1, which binds to MAX and represses MYC transcriptional activity, and through promotion of MYC proteasomal degradation [134, 179]. Indeed, HIF increases MYC phosphorylation at Thr58, triggering MYC ubiquitination, and decreases the de-ubiquitinating enzyme USP28, promoting MYC proteasome-dependent degradation [180]. On the other hand, MYC induces MCM3 and MCM5 proteins [145] which in turn inhibit HIF1 activity by stimulating HIF1α hydroxylation, ubiquitination and degradation [181, 182]. However, prolonged hypoxic conditions reduced MCM mRNA expression in a HIF1-dependent manner, indicating that MCM and HIF1 display antagonistic functions [181, 182]. Interestingly, HIF1 and MYC present sirtuin-mediated shared regulation mechanisms since sirtuin 1 (SIRT1) works in conjunction with both transcription factors while SIRT6 inhibit their transcriptional activity via effects on chromatin [183]. Moreover, dual deficiency of oxygen and glucose suppresses HIF signaling [184] and enhances MYC degradation in cancer cells as an adaptive response to survive under conditions of deficient energy sources [185].

### The PI3K/Akt pathway

Overactivation of phosphoinositide-3-kinase (PI3K)/Akt signaling is commonly observed in human cancers, as it is essential for cell proliferation, growth, survival and metabolic reprogramming [186]. PI3Ks are a family of lipid kinases that integrate prosurvival signals such as growth factors, cytokines, hormones and other environmental cues, translating them into intracellular signals that activate Akt-dependent and Akt-independent downstream signaling pathways [186]. Akt is a serine-threonine protein kinase that is mainly regulated following PI3K activation and through sequential phosphorylation at Thr308 and Ser473 [187, 188]. Since constitutive activation of Akt is frequently found in human tumors, being a central node in the PI3K/Akt signaling pathway, it is potentially interesting to molecularly target components of this pathway for cancer therapy [188].

**Figure 5:**
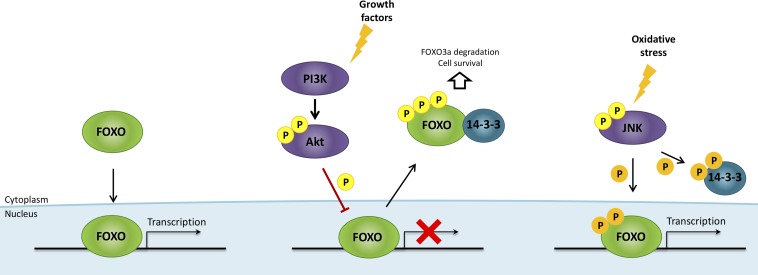
FOXO regulation by growth factors and oxidative stress Growth factors activate PI3K/Akt pathway, resulting in FOXO factors phosphorylation, impairment of FOXO binding activity to DNA and promotion of FOXO interaction with the chaperone protein 14-3-3, which in turn causes FOXO nuclear exclusion, cytoplasmic accumulation and ubiquitin-proteasome pathway-dependent degradation. Oxidative stress activates Jun N-terminal kinase (JNK) signaling, which phosphorylates both FOXO (at other regulatory sites than Akt) and 14-3-3 proteins, triggering the release of FOXO factors, their nuclear translocation and their transcriptional activity. Akt/PBK, protein kinase B; FOXO, forkhead box O; P, phosphate; PI3K, phosphoinositide-3-kinase.

Forkhead box O (FOXO) transcription factors are direct targets of Akt that modulate cell cycle, growth, DNA repair, survival, apoptosis, metabolism, cellular differentiation, resistance to oxidative stress and tumor suppressor pathways [189–193]. Four different FOXO proteins are encoded in mammalian cells; FOXO1, FOXO3a and FOXO4, which are ubiquitously expressed, and FOXO6, which is expressed predominantly in neural cells [194, 195]. Post-translational modifications such as phosphorylation, acetylation and ubiquitination regulate the translocation of FOXO proteins to the nucleus [196] where they activate transcription by binding to gene regulatory regions [197]. The principal mechanism of FOXO transcriptional regulation is FOXO phosphorylation by Akt which impairs its DNA binding activity and promotes its interaction with the chaperone protein 14-3-3. This interaction triggers the nuclear exclusion, cytoplasmic accumulation and ubiquitin-proteasome pathway-dependent degradation of FOXO factors, and promotes cell survival [198, 199]. In the presence of oxidative stress, FOXO proteins are activated and released from 14-3-3 through Jun N-terminal kinase (JNK) signaling [189, 191, 200, 201] (Figure 5).

FOXM1 transcription factor is a crucial regulator of cell proliferation and cell cycle progression that is overexpressed in many types of cancer. Cell differentiation, angiogenesis, senescence, DNA damage repair and tissue homeostasis are regulated by FOXM1, conferring oncogene-like properties to this forkhead subfamily member [202]. Recent studies have reported that FOXO3a represses FOXM1 expression and that they both compete for binding to similar DNA sequences, sharing numerous target genes but being antagonists [196, 203–205]. It is worth noting that FOXO3a and FOXM1 proteins are indirect targets of several conventional and widely used cytotoxic chemotherapeutic drugs such as cisplatin or gefitinib, which mediate their effects through FOXO3a activation and FOXM1 indirect repression via PI3K/Akt signaling pathway inhibition [206–211]. The dysregulation of the PI3K/Akt/FOXO3a axis may lead to drug resistance by enhancing DNA repair, as well as cancer cell maintenance, proliferation and survival through overexpression of FOXM1 [196, 202].

A hallmark of most cancers where the PI3K/Akt pathway is hyperactivated is the inactivation of FOXO proteins [188, 201], postulating FOXO family members as tumor suppressors [212]. Accordingly, PI3K depletion results in FOXO proteins activation, induction of apoptosis, decrease of cell viability and G1 cell cycle arrest with inhibition of CDK4/6, cyclin D and accumulation of p27^Kip1^ [213]. Indeed, *in vivo* models of loss of FOXO function exhibit spontaneous tumor formation, while FOXO overexpression can inhibit tumorigenesis [191, 211, 212, 214–216]. Even though FOXO transcription factors are considered to be tumor suppressors, genetic inactivation of FOXO is not often found in human cancers, being predominantly repressed through overactivation of the PI3K/Akt pathway caused by mutations in *RAS*, *PTEN* or *PI3K* genes [212]. Therefore, the search for compounds that promote activation and relocalisation of FOXO from the cytoplasm to the nucleus is a promising therapeutic approach for cancer treatment and overcoming drug resistance [215, 217].

#### FOXO effects on MYC, HIF1α and mTOR

Activated PI3K/Akt pathway stimulates cell growth and proliferation, and stabilizes MYC through inhibition of GSK3β by preventing MYC phosphorylation at Thr58 [154]. Active PI3K and MYC specifically cooperate in dysregulation of cell growth and proliferation, since both regulate a common set of cellular processes [218]. Conversely, activation of FOXO transcription factors following inhibition of PI3K/Akt signaling represses multiple MYC target genes including those involved in cell proliferation and mitochondrial activity, blocking MYC-mediated cell proliferation and transformation, and reducing ROS production [219, 220]. In addition, FOXO3a induces the expression of the MAD/MXD family of transcriptional repressors, although MXI1 is the only member that is its direct target. Indeed, MXI1 is necessary for efficient inhibition of MYC transcriptional activity [221]. Furthermore, FOXO3a activation considerably reduces MYC protein levels by enhancing phosphorylation of MYC at Thr58, which triggers its proteasomal degradation [220]. Interestingly, FOXO3a-mediated regulation of MYC at different levels enables both acute inhibition of mitochondrial gene expression by MYC degradation and sustained inhibition through MXI1 antagonistic effects [222]. Therefore, the inhibition of the transcriptional activity of FOXO proteins by Akt-mediated phosphorylation is required for MYC-induced cell proliferation and transformation [219].

*FOXO3a* is induced under hypoxic conditions as a direct target gene of HIF1 to mediate the hypoxic repression of nuclear-encoded genes with mitochondrial function by directly antagonizing MYC at their promoters, resulting in reduced mitochondrial mass, oxygen consumption and ROS production [223, 224]. Additionally, FOXO3a promotes cell survival both in hypoxic tumor cells and hypoxic tumor tissue *in vivo*, in contrast with its role as a tumor suppressor under normoxic conditions [212, 223]. On the other hand, FOXO3a prevents HIF1α stabilization by blocking the hypoxia-induced ROS increase [220], and inhibits HIF1α activity through stimulation of CITED2 (Cbp/p300-interacting transactivator 2) expression, also reducing HIF1α-induced apoptosis during hypoxic stress and promoting cell survival [224].

In response to energy stress, FOXO proteins inhibit the mechanistic target of rapamycin complex 1 (mTORC1) signaling through induction of *BCL2/adenovirus E1B 19kDa interacting protein 3* (*BNIP3*) expression, which in turn negatively regulates the mTORC1 activator RHEB and the BCL2 pro-survival family members, resulting in energy stress-induced apoptosis [225]. In addition, mTORC1 inhibition upregulates FOXO3a expression and nuclear accumulation through *FOXO3a* demethylation [226]. Conversely, mTOR complex 2 (mTORC2) phosphorylates the Class IIa histone deacetylases (HDACs) in an Akt-independent manner, resulting in FOXO acetylation, release of MYC proteins from FOXO-mediated repression and the consequent conferral of resistance to PI3K and Akt Inhibitors [227].

**Figure 6:**
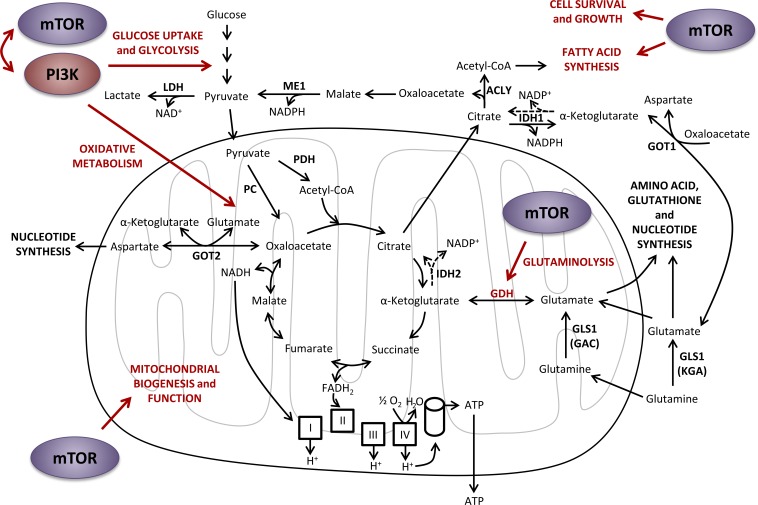
Effects of PI3K and mTOR on central carbon metabolism PI3K and mTOR signaling pathways positively regulate each other's activity, as well as glucose uptake and glycolysis, oxidative metabolism and glutaminolysis. mTOR also promotes fatty acids synthesis and cell survival and growth. ACLY, ATP citrate lyase; ATP, adenosine triphosphate; CoA, coenzyme A; FADH_2_, flavin adenine dinucleotide reduced form; GAC, glutaminase C; GDH, glutamate dehydrogenase; GLS1, glutaminase 1; GOT1, glutamic-oxaloacetic transaminase 1 cytoplasmic form; GOT2, glutamic-oxaloacetic transaminase 2 mitochondrial form; IDH1, isocitrate dehydrogenase cytoplasmic form; IDH2, isocitrate dehydrogenase mitochondrial form; KGA, kidney (K-type) glutaminase; LDH, lactate dehydrogenase; ME1, malic enzyme 1 cytoplasmic form; NAD^+^, nicotinamide adenine dinucleotide oxidized form; NADH, nicotinamide adenine dinucleotide reduced form; NADP^+^, nicotinamide adenine dinucleotide phosphate oxidized form; NADPH, nicotinamide adenine dinucleotide phosphate reduced form; PC, pyruvate carboxylase; PDH, pyruvate dehydrogenase.

### mTOR

The mechanistic target of rapamycin (mTOR, formerly mammalian TOR) is a conserved cytoplasmic serine-threonine protein kinase that acts as a central cell growth regulator by sensing mitogens, energy and amino acids. mTOR pathway regulates cell survival and growth through modulation of some pivotal cellular processes including protein synthesis, ribosome biogenesis, autophagy and metabolism [228]. In fact, the dysregulation of mTOR-dependent cellular homeostasis maintenance is associated with several human diseases such as cancer and considerable research efforts have been made to efficiently inhibit mTOR signaling [229, 230].

mTOR forms two functionally and structurally different multiprotein complexes named mTOR complex 1 (mTORC1) and 2 (mTORC2). mTORC1 activity is regulated by growth factors, oxygen and nutrient availability. Activation of PI3K/Akt and RAS/RAF/ERK pathways by growth factors results in Akt- and ERK-mediated phosphorylation and inactivation of the heterodimer tuberous sclerosis 1 (TSC1)/TSC2, which is a GTPase-activating protein (GAP) that negatively regulates mTORC1 through inhibition of the RAS homolog enriched in brain (RHEB) GTPase [231]. Remarkably, intracellular amino acids are necessary for the activation of mTORC1 since they activate the mechanism by which mTORC1 is able to interact with and be activated by RHEB [67].

Activated mTORC1 signaling cascade initiates with the direct phosphorylation of the regulators of translation eukaryotic translation initiation factor 4E (eIF4E)-binding protein 1 (4E-BP1) and S6 kinase 1 (S6K1), which promote protein synthesis [232]. In addition, mTORC1 regulates lipid homeostasis through activation of the transcription factors sterol regulatory element-binding protein 1/2 (SREBP1/2), which in turn control the expression of genes involved in fatty acid, triglyceride, phospholipid and cholesterol synthesis [228, 233]. Interestingly, mTORC1 also promotes mitochondrial biogenesis and the expression of genes involved in oxidative metabolism [234, 235]. On the other hand, mTORC2 pathway regulation and function remain poorly understood. mTORC2 signaling is independent of nutrient availability but is sensitive to PI3K signaling [228]. mTORC2 directly activates Akt through phosphorylation of the Ser473 residue, which in turn activates mTORC1, both situating mTOR upstream and downstream of Akt [236]. It is worth noting that acute rapamycin treatment specifically inhibits mTOR when it is part of mTORC1 but not of mTORC2 [228, 229].

#### mTOR regulation by hypoxia and MYC

Hypoxic oxygen levels inhibit mTORC1 by activating the TSC1/TSC2 complex through two different pathways. On the one hand, hypoxia reduces cellular ATP levels and triggers 5′-AMP-activated protein kinase (AMPK) activation, which positively regulates TSC1/TSC2 in a HIF1-independent manner [237]. On the other hand, hypoxia activates TSC1/TSC2 by the transcriptional induction of *regulated in development and DNA damage responses 1* (*REDD1*) gene, antagonizing other pathways that promote growth through TSC1/TSC2 inhibition via Akt [238, 239]. Hypoxia can also negatively regulate mTORC1 through the hypoxia-inducible protein BNIP3 binding to RHEB, which inhibits the ability of RHEB to activate mTORC1 [240]. Conversely, MYC acts as a strong and direct repressor for *TSC2* expression by binding to its promoter, resulting in mTORC1 activation [241]. In addition, mTORC1 downstream effector S6K1 phosphorylates the eukaryotic initiation factor eIF4B, enhancing MYC translation efficiency and positively regulating glutaminase (GLS) and glutamate dehydrogenase (GDH) [101, 242]. Moreover, glutaminolysis and α-ketoglutarate production, in response to glutamine and leucine [68], also mediate mTORC1 activation [111].

In summary, mTOR, PI3K, HIF and MYC are key regulators of cellular metabolism that are frequently altered in cancer, collaborating in both synergistic and antagonistic ways. A better understanding of the relationship between these pathways as well as the identification of other key players in the regulation of the tumor metabolic reprogramming are fundamental challenges for the development of new strategies for cancer treatment.

## THERAPEUTIC PERSPECTIVES

### Targeting metabolic reprogramming in cancer therapy

Development of malignancy is accompanied by a complete metabolic reprogramming closely related to the acquisition of most of cancer hallmarks [2, 28]. Many known genetic and epigenetic alterations converge in a common adaptation of tumor cell metabolism [30]. Indeed, metabolic properties of tumor cells are significantly different from those of non-transformed cells. In addition, tumor metabolic reprogramming is linked to drug resistance in cancer treatment [243, 244]. Accordingly, metabolic adaptations are also involved in different therapeutic approaches for cancer therapy. It is worth noting that some of the first chemotherapeutical agents used in cancer treatment were antimetabolites, such as aminopterin, methotrexate or 5-fluorouracil, that impaired the nucleotide synthesis and DNA replication [245, 246]. From then on, numerous metabolic pathways and enzymes have been successfully tested as anticancer targets [247]. Since aerobic glycolysis is one of the key metabolic features of cancer cells, many studies are focused on inhibiting this pathway by blocking the enzymes that control it [248]. Targeting the PPP with dehydroepiandrosterone (DHEA) and oxythiamine to respectively inhibit G6PD and TKT has also proven to have antitumor effects [50, 244, 249]. Interestingly, promoting pyruvate dehydrogenase (PDH) activity with dichloroacetate (DCA) presents promising results with minor side effects in early phase clinical trials with glioblastoma patients by suppressing angiogenesis, increasing mitochondrial ROS, inducing apoptosis, blocking HIF1 signaling and activating tumor suppressor p53 [250–252]. In fact, DCA inhibits PDHK leading to the metabolic switch from glycolysis to oxidative phosphorylation through PDH reactivation [250]. Moreover, combined therapies with DCA and conventional cancer therapeutics such as omeprazole and tamoxifen show synergistic antitumor effects which can overcome drug resistance [253]. Ongoing clinical trials with DCA as a single agent or in combination with other therapeutics are being conducted for patients with recurrent or metastatic solid tumors and head and neck carcinoma (clinical trials NCT00566410 and NCT01386632).

There is a growing interest on the development of pharmacological strategies to inhibit tumor glutamine metabolism. The use of amino acid analogues such as acivicin led to severe side effects in clinical trials, aiming for more selective therapeutic strategies [254]. As a result, GLS1 isoform has emerged as a promising target for cancer therapy and several specific small molecule inhibitors of GLS1 have recently been characterized. Compound 968 and BPTES (bis-2-(5-phenylacetamido-1,2,4-thiadiazol-2-yl)ethyl sulfide) are two allosteric inhibitors of GLS1 that exhibit antitumor activities in numerous pre-clinical studies and several tumor types [100, 108, 121, 255–257]. Remarkably, the selective inhibitor of GLS1 known as CB-839 presents *in vitro* antiproliferative activity against acute myeloid leukemia cells [258], and a panel of triple-negative breast cancer cell lines, but not estrogen receptor (ER) or human epidermal growth factor receptor 2 (HER2) positive cell lines, as well as *in vivo* efficacy in breast cancer xenograft models [107]. In addition, CB-839 has recently entered phase I clinical trials without displaying central nervous system toxicity (clinical trials NCT02071862 and NCT02071888). In fact, GLS1 inhibition is a good strategy for tumor cells that overexpress MYC and thus present glutamine dependence [22, 99]. It is worth mentioning that, to date, no effective MYC inhibitors have been developed despite the fact that MYC overexpression is frequently found in human cancers [125, 259–261]. However, targeting GLS1 significantly antagonizes the growth of tumors presenting MYC overexpression and can be exploited as a novel antitumor therapy [100].

### Combination therapies

The identification of cytotoxic compounds has led the development of antitumor therapeutics until in the recent years chemotherapy advanced into the era of molecularly targeted therapeutics [262]. The bases of molecular targeted cancer therapy are to selectively kill tumor cells while sparing non-malignant cells, and prevent tumor resistance emergence and relapse [263]. However, solid tumors response to targeted monotherapy is limited and frequently associated with the development of drug resistance. In addition, the design of targeted therapies requires the definition of the activated oncogenic pathways in transformed cells and the availability of selective small-molecule inhibitors directed to these pathways. The modest efficacy of current therapies is also caused by the high degree of tumor clonal and genetic heterogeneity, since inhibition of a single target does not necessarily eradicate the tumor. Therefore, the use of combination therapies of selective agents and/or cytotoxic agents that inhibit two or more molecular targets in a single pathway, or in parallel or compensatory pathways, is an attractive strategy for cancer treatment [264]. Additionally, the simultaneous inhibition of multiple targets or redundant pathways is aimed at improving treatment efficacy and overcoming and/or preventing the emergence of adaptive resistance.

Then, in order to select appropriate molecular targets for inhibition or modification, is necessary to first perform a tumor expression profiling to identify its specific oncogenic signatures, and confirm that the target is tumor specific, non-redundant, and able to influence the outcome of tumor progression [263, 265]. However, many oncogenic pathways cannot be directly targeted with small-molecule inhibitors [260]. Remarkably, gene expression analysis can be used as a predictive tool to identify the oncogenic pathways which are dysregulated in a specific tumor, providing a potential basis for guiding the use of pathway-specific drugs and directing combination therapies aimed to slow tumor growth and progression, improve treatment response, and overcome therapeutic resistance [265].

## CONCLUDING REMARKS

The understanding of cancer cell biology is of pivotal importance to identify biomarkers for early diagnosis and design new therapeutic strategies. In particular, tumor cells switch their core metabolism to meet the increased requirements of cell growth and division. Accordingly, oncogenic signals converge to reprogram tumor metabolism by enhancing key metabolic pathways such as glycolysis, PPP, glutaminolysis and amino acid, lipid and nucleic acid metabolism [19, 63]. Therefore, tumor metabolic reprogramming is a direct result of the re-engineering of intracellular signaling pathways that are altered by activating mutations in oncogenes, loss-of-function mutations in tumor suppressor genes and epigenetic modifications, which finally gives to transformed cells a proliferative advantage over non-malignant cells [5, 6]. However, the final tumor phenotype depends on the homeostatic nature of metabolism, since metabolic rewiring is associated with compensatory and regulatory adjustments. There are several levels of metabolic adaptations, from changes in the concentrations and fluxes associated with substrate-enzyme affinities to adjustments coordinated by metabolite-based regulatory loops or determined by changes in the activity of regulatory proteins (e.g. MYC and HIF). In fact, metabolic rewiring allows tumor cells to exhibit rapidly adaptive responses to changes in tumor microenvironment, promoting tumor progression and acquired resistance to targeted therapeutics [266, 267]. On the other hand, tumor metabolic adaptation after single-agent treatment can reveal new cell dependences and vulnerabilities which, in turn, may be potential candidates to be targeted in combination therapies. The final aim of the combination treatments is to achieve synergistic therapeutic effect, dose and toxicity reduction, and to minimize or delay the induction of drug resistance [268].

Personalized medicine in cancer requires the correct diagnosis of cancer to give patients the most appropriate treatment according to their individual circumstances and the molecular characteristics of their tumors. However, personalized medicine is still at a relatively early stage in its development, and therefore the classification of cancers is based on critical molecular targets identified by translational research. Hence, more efforts should be put into understanding the tumor biology in order to identify the involved targets and determine the optimum treatment for each specific tumor. Likewise, the developing of new therapeutic strategies that specifically target the molecular pathways involved in promoting tumor cell proliferation and survival, such as targeted therapies, is a major focus of cancer research today [263]. However, the currently available chemotherapeutic treatments exhibit modest efficacy due to their side effects and drug resistance [267]. In addition, the design of more efficient targeted therapies requires a better definition of the activated oncogenic pathways in transformed cells and the availability of selective small-molecule inhibitors directed to these pathways. In this context, the search for combined chemotherapies with low systemic toxicity that inhibit two or more molecular targets in a single pathway, or in redundant or compensatory pathways, is a promising strategy for cancer treatment [264]. With this purpose, both metabolic tumor characterization and gene expression analysis can be used to identify the dysregulated molecular pathways in a specific tumor, providing a potential basis for guiding the use of target-specific drugs and directing combination therapies [265].
